# Supplementary material for: AI-assisted Cryo-ET workflow for 3D visualization of chromatin during cellular differentiation
Source: Biochem Biophys Rep. 2026 Sep 11;48:102789. doi: 10.1016/j.bbrep.2026.102789 (PMC13584007; doi:10.1016/j.bbrep.2026.102789)
Supplement: Supplementary file 1 [file mmc1.docx]

**Supporting Information for;**

**AI-Assisted Cryo-ET Workflow for 3D Visualization of Chromatin during Cellular Differentiation**

*John Watt^1^, Cullen Roth^2^, Isaac Huber^1^, Alexander S. Hall^3^, Manish Kumar Singh^1*^, Sofiya Micheva-Viteva^2^, Karissa Y. Sanbonmatsu^4^_,_ Shawn R. Starkenburg^2^, Christina R. Steadman^2^*

1. Materials Physics and Applications Division, Los Alamos National Laboratory, Los Alamos, NM 87545, USA

2. Bioscience Division, Los Alamos National Laboratory, Los Alamos, NM 87545, USA

3. Thermo Fisher Scientific, Houston, TX 77080, USA

4. Theoretical Division, Los Alamos National Laboratory, Los Alamos, NM 87545, USA

^*^ Now at Department of Physics, University of Allahabad, Prayagraj, UP 211002, India

Figure S1. Identification of chromatin ‘domains’ using Image J and Thermo Scientific Avizo^TM^ 3D Pro 2022.2 and 2023.1 software.
